# Long-term clinical results of using a posteromedial all-inside and anteromedial inside-out approach to repair unstable or irreducible bucket-handle medial meniscal tears

**DOI:** 10.1186/s10195-023-00691-w

**Published:** 2023-04-06

**Authors:** Sohrab Keyhani, Mohammad Movahedinia, Robert F. LaPrade, Mohamad Qoreishy, Fardis Vosoughi

**Affiliations:** 1 Akhtar Orthopedic Hospital, Shahid Beheshti University of Medical Sciences, Sharifi Manesh Street, Shariati Street, Tehran, Iran; 2 Department of Orthopedic Surgery, University of Minnesota, Minneapolis, USA; 3 Department of Orthopaedic and Trauma Surgery, Shariati Hospital, Tehran University of Medical Sciences, Tehran, Iran

**Keywords:** Bucket-handle medial meniscal tear, Chronic, Unstable, Posterior arthroscopy, All-inside, Provisional fixation

## Abstract

**Background:**

In irreducible bucket-handle medial meniscal tears (BHMMTs), the displaced central fragment is rotated 180° or more, meaning reduction is impossible without derotation of the tear. In chronic cases, the rotated meniscus is deformed and degenerated; thus, the issue of reproducibility and repairability arises. This study presents the clinical outcomes of chronic irreducible and unstable locked BHMMTs. Radiological outcomes were also evaluated using magnetic resonance imaging (MRI).

**Methods:**

This is a retrospective study of 37 patients with 11 cases of irreducible BHMMT and 26 cases of reducible but unstable chronic BHMMT who underwent operations between 2011 and 2016. Posterior arthroscopy was performed after temporary meniscus fixation using a provisional needle fixation technique. After the posterior repair with vertical mattress sutures was completed using an all-inside technique, the classic outside-in technique was performed for the anterior third of the meniscus. The Lysholm, International Knee Documentation Committee (IKDC), and Tegner activity scores were obtained from all patients before surgery and at the latest follow-up. Radiological evaluations were performed using MRI before surgery and at 6 months postoperatively.

**Results:**

Between 2011 and 2016, a total of 37 consecutive patients with irreducible and chronic BHMMTs underwent surgery. The average postoperative follow-up was 7.2 ± 1.4 years (mean ± SD). Postoperative Lysholm (89.57 ± 2.7) and IKDC (87.22 ± 3.2) scores improved significantly at the last follow-up when compared with the pre-operative scores (38.44 ± 4.5 and 23.52 ± 7.8, respectively). According to the Tegner activity scale, patients’ postoperative activity levels remained unchanged compared to preoperative levels at the last follow-up.

**Conclusion:**

Posterior knee arthroscopy with the all-inside posterior suture and inside-out anteromedial suture technique presented in this study yielded excellent clinical outcomes when used to repair chronic irreducible or unstable BHMMTs.

*Level of evidence*: Level IV.

**Supplementary Information:**

The online version contains supplementary material available at 10.1186/s10195-023-00691-w.

## Introduction

The importance of preserving the meniscus, especially for preventing degenerative changes, has been documented [1, 2]. Meniscal repair is considered the treatment of choice for medial meniscus injuries whenever possible [3, 4]. A bucket-handle medial meniscal tear (BHMMT) can involve the majority of the medial meniscus and resection is associated with significant morbidity [5]. Insufficient repair of a BHMMT significantly lowers knee function and leads to debilitating symptoms in the patient [6].

The complexity and size of a BHMMT are two important factors that predict potential failure [7]. Other factors include concomitant anterior cruciate ligament (ACL) reconstruction and the chronicity of the tear [8, 9]. The reducibility of BHMMTs has been assessed subjectively as either easy, moderate, or irreducible based on Lim et al.’s classification method [10]. In irreducible cases, the displaced central fragment is rotated 180° or more, meaning that reduction is impossible without derotation. In chronic cases, the rotated meniscus is deformed and degenerated; thus, the issue of instability arises [11]. Although reduction is sometimes possible in chronic cases, the reduced and deformed fragment does not remain in the anatomical position and is immediately displaced into the notch, making repair impossible [11].

In the present study, we repaired several chronic irreducible or unstable locked BHMMTs. The technique utilized included provisional percutaneous needle fixation viewed from the anterior portals to maintain the reduction, and then an all-inside repair using a modified posterior knee arthroscopy with a posterolateral trans-septal view. This study reports the clinical outcomes of these cases during a mid-term clinical follow-up.

## Material and methods

**Fig. 1:**
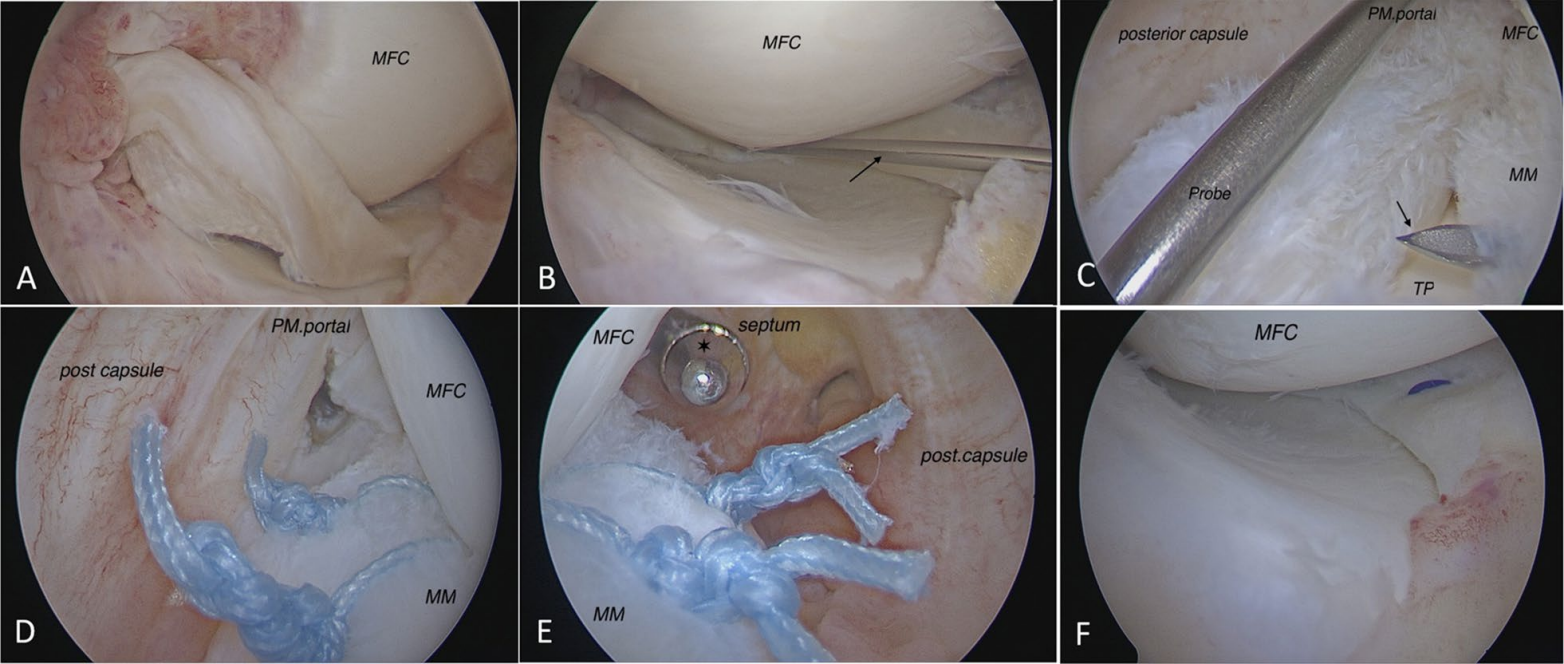
**A** Locked unstable medial meniscus in an anterolateral portal view of a right knee. Anterolateral portal (**B**) and posterolateral trans-septal (**C**) views show the torn medial meniscus fixed using a needle (*arrow*) percutaneously. Posterolateral trans-septal (**D**), posteromedial (**E**), and anterolateral portal (**F**) views after meniscal repair. *MM* medial meniscus, *TP* tibial plateau, *MFC* medial femoral condyle, * arthroscopic sheath, *PM.portal* posteromedial portal

This is a retrospective study of a total of 37 consecutive patients with an average age of 43 years (32–55 years) who were treated with a modified posterior arthroscopic technique to repair irreducible or unstable locked BHMMTs from 2011 to 2016. Approval was acquired from the local ethics committee, and all participants filled out a written informed consent form before their operations. Inclusion criteria were patients aged 55 years or younger with a chronic post-traumatic locked BHMMT in the red-red or red-white zone and a concomitant ACL tear. Chronicity was defined as an interval of more than 12 weeks between the history of the last episode of locking and the surgery [10]. BHMMTs were limited to reducible but unstable locked menisci (Fig. 1A) and irreducible menisci with a complete rotation (Lim et al.’s [10] classification type 3) (Fig. 4C). Exclusion criteria were any other intra-articular surgery or revision surgery, which did not include any of the participants. Furthermore, all stable and reducible BHMMTs were excluded. All patients had a concomitant ACL tear and were operated on by the senior author, and all arthroscopic procedures were recorded. Patient information was extracted from medical records and the hospital PACS system.

Magnetic resonance imaging (MRI) was performed preoperatively and 6 months postoperatively for all patients. Lysholm, International Knee Documentation Committee (IKDC), and Tegner activity scale scores were obtained from all patients before surgery. Patients were asked to score themselves on the Tegner activity scale based on their condition before suffering their injury. At 5–10 years (an average of 7 years) after the operation, these patient-reported outcomes were assessed again, and patients were clinically evaluated for meniscal repair failure. Failure was defined as the presence of at least one of Barrett et al.’s [12] criteria, including joint effusion, pain along the medial joint line, a positive provocation test, and the need for meniscectomy. Complete healing was assumed if none of the criteria were met [13].

### Surgical technique

Patients were placed in the supine position, and the affected limb was positioned over the edge of the operating table to allow access to either side of the knee, while the contralateral leg was placed in a lithotomy position. A high-thigh tourniquet was used. Standard anterior portals were used for routine diagnostic arthroscopic visualization and trans-portal ACL reconstruction, and a 30° lens was used throughout the surgery. First, the semitendinosus autograft was acquired, and the femoral closed socket tunnel was reamed. The meniscal repair process was then carried out.

**Fig. 2:**
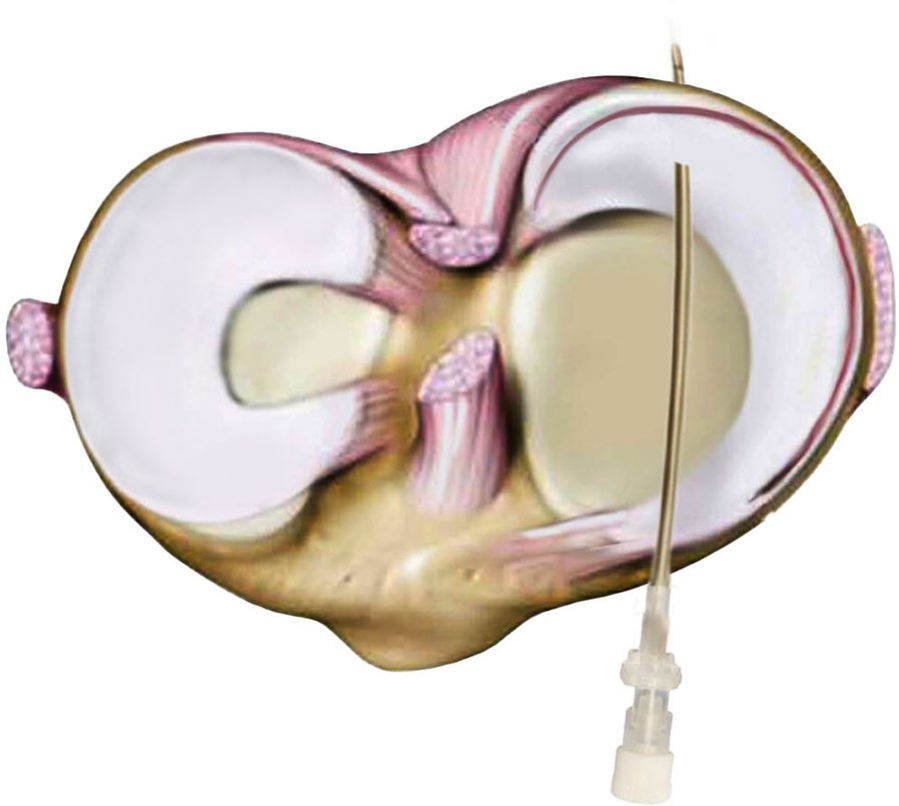
Percutaneous provisional fixation in a right knee using a prebent needle inserted from anterior to posterior, perpendicular to the meniscal tear

Using the anterior portals, the adhesion bands of neighboring structures inside the notch were removed to mobilize the meniscus, and tear borders were refreshed in situ with a shaver before the reduction. With the patient’s knee positioned in 30° of flexion with valgus force, the meniscus was reduced by gentle manipulation using an arthroscopic trocar through an anteromedial portal. In the case of complete meniscal rotation (Lim et al.’s [10] classification type 3), a grasper was used through an accessory high anterolateral portal to derotate the fragment. The technique of reducing the torn meniscus depends on the skill of the surgeon and can easily be done using a trocar or grasper. For a better understanding, refer to the technique shown in Additional file 1: Video S1 of this article. Then, a 16-gauge needle was used as a percutaneous provisional fixation; this needle was inserted from anterior to posterior, perpendicular to the meniscal tear, to keep the reduced meniscus in the anatomical position (Fig. 1B and 2). This needle acted like a reduction in–out suture to fix the posteromedial part of the meniscus between the posterior and the middle-third parts at the beginning of the meniscal repair process (Fig. 1C).

To maintain better stability, the needle was slightly bent and then passed over the anterior horn of the meniscus and inserted into the posterior horn (Fig. 2). Usually, one needle was sufficient to perform this task, but more needles were sometimes required in cases of gross instability. The needle was removed after completion of the posterior meniscal repair.

**Fig. 3:**
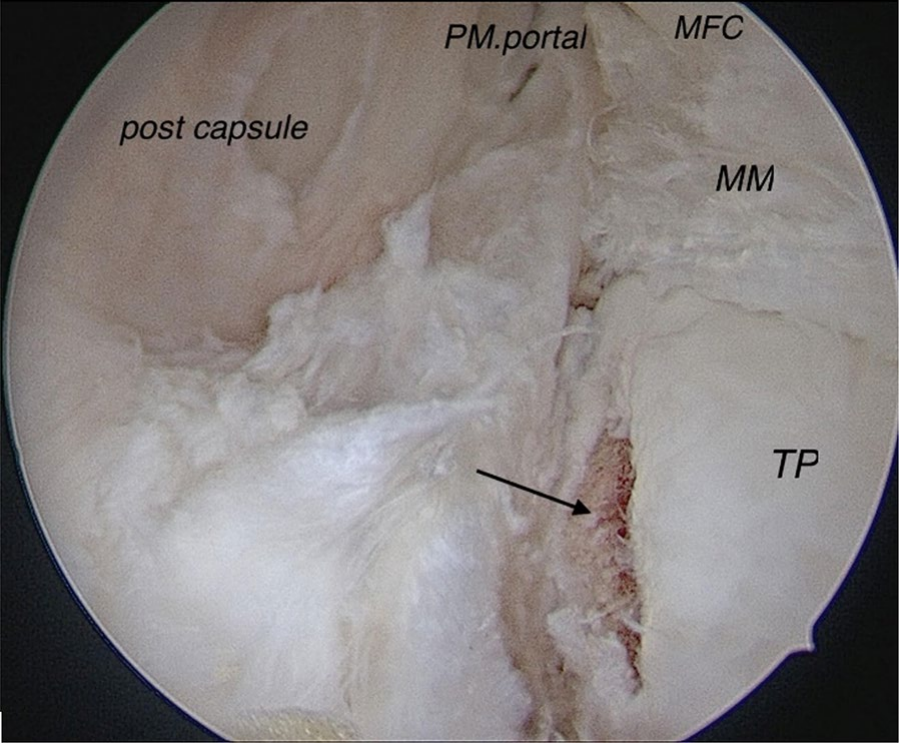
The posteromedial non-cartilaginous rim of the tibial plateau below the torn meniscus was abraded using a bur to reach the cancellous bleeding bone. *MM* medial meniscus, *TP* tibial plateau, *MFC* medial femoral condyle, *PM.portal* posteromedial portal

After temporary meniscus fixation, posterior arthroscopy was performed following Keyhani et al.’s method [14], and the posteromedial and posterolateral trans-septal portals were created for repair and assessment, respectively. Medial collateral ligament (MCL) pie-crusting was not necessary to improve the visualization and reduction of the meniscus. Healing was enhanced by performing complete abrasion of the posterior meniscal tear borders with a shaver or meniscal rasp through the posteromedial portal. The posteromedial non-cartilaginous rim of the tibial plateau below the torn meniscus was abraded using a bur to reach the cancellous bleeding bone (Fig. 3). Once the tourniquet was released, these in-situ blood clots were confined to the undersurface of the meniscus to promote healing.

Under direct vision through the posterolateral trans-septal portal, a suture hook (Lasso ConMed-Linvatec, Utica, NY, USA) loaded with No. 1 PDS (polydioxanone suture; Ethicon, Somerville, NJ, USA) was passed through the peripheral and central segment of the medial meniscus. The posteromedial portal was used as a working portal, and a shuttle relay system was used to lift the peripheral fragment to the level of the central fragment to increase the contact surface for the repair process. The PDS was replaced with No. 2 fiber wire (Arthrex) and, finally, an SMC sliding knot was applied with a knot pusher. Suturing was repeated every 5 mm and terminated at the posterior corner point [15] (Fig. 1D–E).

After the posterior meniscal repair using an all-inside technique was completed, the classic outside-in technique was performed for the anterior third of the meniscus using anterior portals and No. 1 PDS (Fig. 1F). Sutures were placed anterior to the MCL to prevent MCL irritation, and abrasion was only done with a shaver for the mid-third of the meniscus.

The stability of the meniscus was re-evaluated with a probe at the end of the operation. Finally, the tibial tunnel was reamed, and a quadrupled semitendinosus autograft was used for a single-bundle anatomical ACL reconstruction. An endobutton or screw was used for fixation on the femoral side, and double fixation with a screw and ENDOTACK (KARL, STORZ) was used on the tibial side.

### Rehabilitation

Toe-touch weight bearing was allowed for the first 2 weeks after the operation, while the knee was immobilized by an extension brace with an allowed passive range of motion (ROM) of up to 45°. During the third and fourth weeks, partial-weight-bearing ambulation and flexion with a ROM of 90° were allowed. Full-weight-bearing ambulation and full ROM were allowed only after 8–12 weeks after the operation.

### Statistical analysis

SPSS software was used to analyze the data. Lysholm and IKDC scores (parametric variables) before and after the operation were compared using the paired* t*-test. The Tegner activity scale scores before the injury and after the operation were compared using the Wilcoxon test (non-parametric variable). The significance threshold was* P* < 0.001.

## Results

Thirty-seven study patients had 11 cases of irreducible BHMMT (Lim et al.’s [10] classification type 3) and 26 cases of reducible but unstable chronic BHMMT that needed provisional fixation with a needle before the repair process could begin. In total, 28 males and 9 females with a mean age of 29.5 ± 3.5 years at the time of the operation participated in the study. The mean time interval from an ACL injury to the first episode of locking was 13.40 ± 2.50 and 15.44 ± 1.54 months for patients with irreducible or unstable chronic BHMMTs, respectively. Surgery was performed 23.65 ± 5.33 and 35.45 ± 7.12 months after ACL tears for the irreducible or unstable chronic BHMMT cases, respectively. The latest follow-up was carried out 5–10 years after the surgery (7.2 ± 1.4 years, mean ± SD).

Lysholm and IKDC scores improved significantly at the last follow-up when compared with the pre-operative scores (Table 1). Based on the post-operative Tegner activity scale scores (6.75 ± 0.4), patients were able to return to pre-injury activity levels (6.8 ± 0.12). There were no intraoperative findings of articular cartilage damage in any of the participants. After the reduction, a ramp lesion was seen in all patients.

**Table 1:** IKDC and Lysholm scores of the patients before the operation and at the last follow-up

Clinical score	Pre-operation (mean ± SD)	Last follow-up (mean ± SD)	*p* value
IKDC^a^	23.52 ± 7.8	87.22 ± 3.2	< 0.001
Lysholm	38.44 ± 4.5	89.57 ± 2.7	< 0.001

^a^International Knee Documentation Committee

**Fig. 4:**
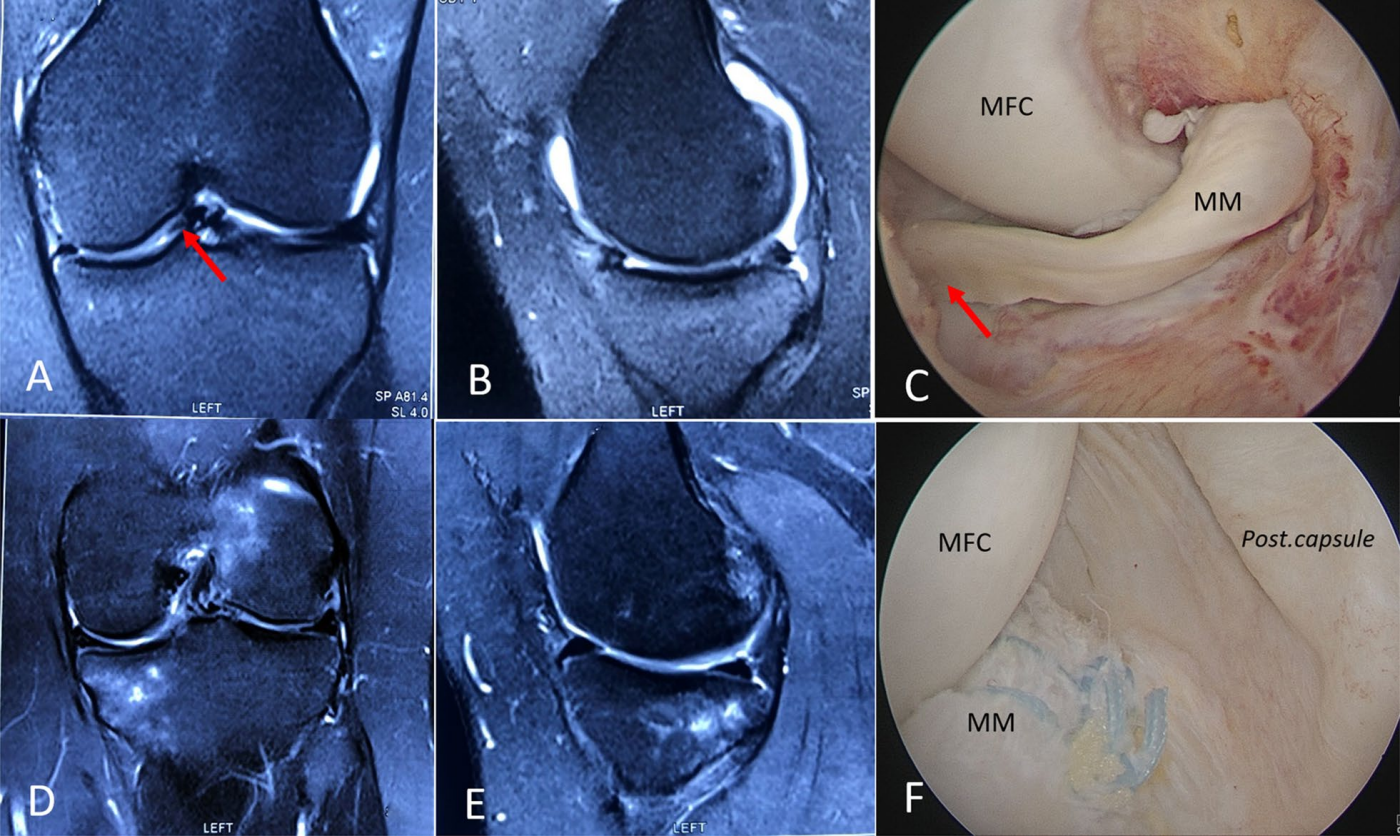
Preoperative T2-sequenced coronal (**A**) and sagittal (**B**) MR images of an unstable chronic BHMMT (*arrow*). **C** Rotation (*arrow*) is visible in an anterolateral portal view of the left knee. Postoperative coronal (**D**) and sagittal (**E**) MR images show healing after 6 months. Posterolateral trans-septal view (**F**) after meniscal repair shows anatomical reduction. *MR* magnetic resonance, *MFC* medial femoral condyle, *MM* medial meniscus

**Fig. 5:**
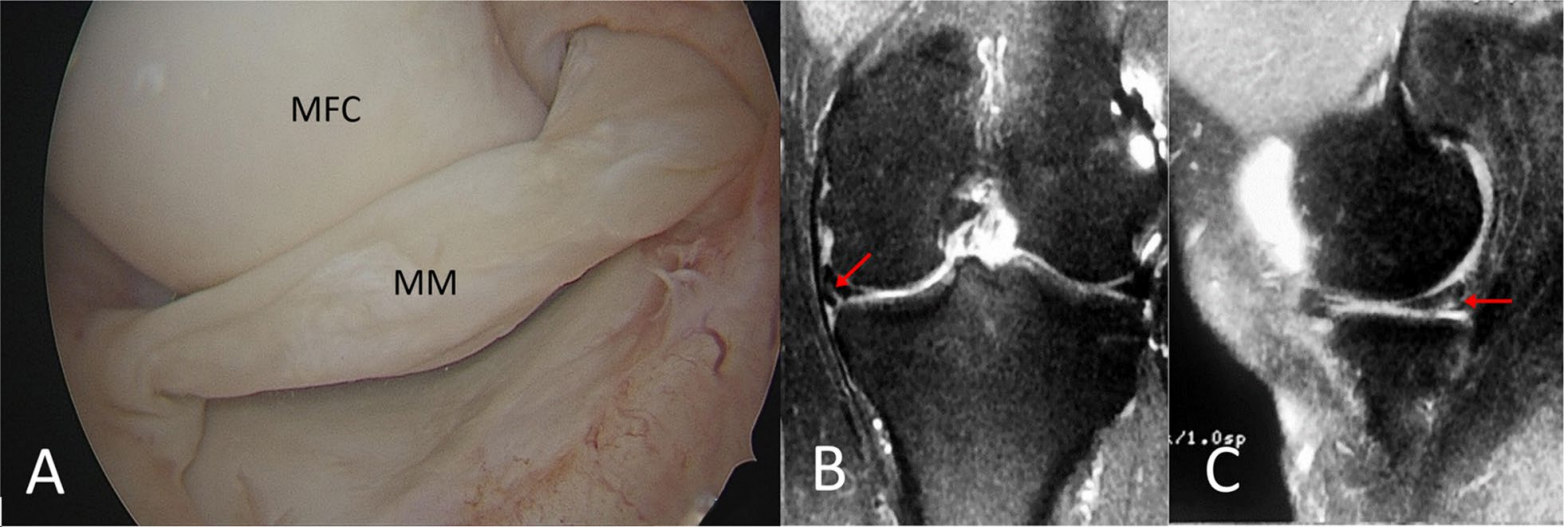
**A** Intraoperative arthroscopic image of a left knee showing an irreducible BHMMT (Lim et al.'s classification type 3). Postoperative T2-sequenced coronal (**B**) and sagittal (**C**) MRI shows an unhealed repair (*red arrow*) after 6 months. *MRI* magnetic resonance imaging, *MM* medial meniscus, *MFC* medial femoral condyle

According to the MRI findings, 36 participants experienced a reduced meniscus with sufficient healing during the sixth month after surgery (Fig. 4). Only one patient with an irreducible BHMMT showed no sign of healing according to an MRI scan performed 6 months after surgery, but the repaired meniscus was in the reduced position and without any symptoms (Fig. 5). This patient had a 15° extension deficit, which was not resolved by physiotherapy (the patient refused a second-look arthroscopy). Patients were able to return to sport-specific activities 9–12 months (average of 10.6 months) after surgery once a full, pain-free ROM was achieved and the muscle strength of the injured limb was equal to that of the contralateral limb.

## Discussion

The most important finding of this study was that the technique described in this study is an effective treatment for unstable or irreducible BHMMTs. All patients reported complete clinical healing 5–10 years postoperatively, which is in line with Barrett et al.’s criteria. Radiological healing was also acceptable in all but one patient 6 months after surgery. The stable repair technique performed in this study allowed the surgeon to begin early knee ROM up to 45° for all patients. It is reported that other techniques do not allow early knee ROM until during the first 2 weeks after surgery [11, 12, 16, 17].

A BHMMT often begins in the posterior horn of the meniscus and spreads through the middle-third junction to the anterior third [9]. Arthroscopic repair of BHMMTs is difficult due to their complex pathology. Previous estimates indicate that BHMMT repairs fail 14.8% of the time [6]. Better prognoses have been reported for repairing acute meniscal tears than chronic injuries [10, 15]. However, recent studies suggest that good and even excellent results are obtained for chronic lesions [17, 18]. Reducing the BHMMT and reducing it in place during the repair process are challenging but essential steps for successfully repairing this type of injury [19, 20].

Anterior portal arthroscopy has limitations when repairing locked BHMMTs because of the narrow medial compartment of the knee. MCL trephination is often necessary to create more space [21, 22]. Iatrogenic chondral lesions and residual unrepaired tears are morbidities that prevent symptoms from being adequately relieved, thus leading to higher failure rates [11]. It is difficult to visualize the posterior horn of the medial meniscus with classic anterior portals, which may result in the blind placement of sutures and insufficient debridement of the chronic meniscal tear borders [15].

Recently, posterior knee arthroscopy has gained attention in meniscal repair because of its direct access to meniscal borders [23]. Posterior portals minimize the risk of cartilage and instrument damage and the need for MCL trephination to create extra space [13, 24]. In this study, posterior portals were used to enable meniscal posterior border abrasion and fibrous tissue removal. Freshening the meniscal borders was the first step toward meniscal repair [8, 24], and posterior arthroscopy facilitated vertical mattress suture placement with high biomechanical strength to allow contact surface instead of point contact repair [11, 16, 25].

After the reduction, a ramp lesion was seen in all patients, supporting the theory that BHMMTs can initiate from a posterior ramp tear. This result highlights the importance of routine assessments of the posterior compartment during ACL reconstruction so as not to miss a ramp tear. Following this theory, the direct view of the posterolateral trans-septal portal helped us to initiate the repair process from the ramp area, adjacent to the meniscus root attachment, where the tears originated. Posterior arthroscopy also helped to elevate the peripheral depressed capsule, which resulted in greater anatomic reduction and a larger contact surface area [26].

In the presented repair technique, posterior meniscal repair with all-inside sutures every 5 mm and the repair of the anterior third eliminated the need for suturing in the middle third of the meniscus. We simply abraded the mid-third of the meniscus with a shaver, keeping the MCL intact and decreasing the risk of arthrofibrosis due to MCL irritation. We also avoided applying reduction sutures through the posteromedial capsule, which is often performed in conventional inside-out repairs that require additional skin incisions with an increased risk of neurovascular injuries [27]. Avoiding MCL entrapment as well as safe knot placement to prevent articular cartilage irritation are additional possible reasons for the favorable results reported in this study [26].

Under direct vision through the posterolateral portal, the entire posterior part of the meniscus was accessible from the root attachment to the posteromedial corner, which allowed us to apply all-inside vertical mattress sutures. Because vertically orientated and full-thickness sutures are used in this technique, the circumferential meniscus fibers act as a single thick fiber and provide surface contact, providing higher torsional strength and better healing [20]. In addition to all the abovementioned benefits, the use of the all-inside technique also reduces the cost of surgery.

Alabi et al. [16] emphasized the importance of applying surface contact instead of point contact sutures when repairing BHMMTs. They used an inside-out technique for the anterior two-thirds and an all-inside technique for the posterior third, utilizing anterior portals. They also employed an MCL release to repair the posterior third. Tan et al. [28] used a reduction suture in the mid-third of the meniscus to repair the posterior portion through the anterior portals using an all-inside technique. Like Alabi et al., they initiated the repair process from the anterior tear margin. Such reductions may prevent anatomic reduction of the posterior third of the meniscus and require a posteromedial incision. In the presented technique, the repair process initiated from the posterior tear region, where the tear originated.

Researchers have described techniques for temporarily stabilizing meniscal fragments using a probe or trocar [16, 28]. These techniques require the use of up to three portals during arthroscopy, and they could result in a loss of reduction during the repair process. The provisional needle fixation technique described in this study does not require a second surgeon and provided secure reduction during the repair process.

It is recognized that there were some limitations to this study. Given that some cases of incomplete radiological healing may progress to complete healing over time [12], the lack of a repeat MRI at the final follow-up was one of the limitations of this study. Our study design was also limited by its retrospective design. Using a control group in the future could allow researchers to more accurately assess the effectiveness of the technique presented in this study. Finally, the all-inside suture technique used in this study was technically demanding, which makes it relatively unpopular among surgeons.

## Conclusion

Posterior knee arthroscopy with the all-inside posterior suture and inside-out anteromedial suture technique presented in this study yielded excellent clinical outcomes when used for the repair of chronic irreducible or unstable BHMMTs.

## Supplementary Information

**Additional file 1.** Posteromedial all-inside and anteromedial inside-out approach to repair unstable or irreducible bucket-handle medial meniscal tears.

## Data Availability

The datasets used and/or analyzed during the current study are available from the corresponding author on reasonable request.
